# Effectiveness of β-blockers in improving 28-day mortality in septic shock: insights from subgroup analysis and retrospective observational study

**DOI:** 10.3389/fcvm.2024.1438798

**Published:** 2024-09-03

**Authors:** Ling Zhang, Yue Yu, Tong Wu, Tingting Pan, Hongping Qu, Jingyi Wu, Ruoming Tan

**Affiliations:** Department of Critical Care Medicine, Ruijin Hospital, Shanghai Jiao Tong University School of Medicine, Shanghai, China

**Keywords:** septic shock, β-blockers, hemodynamics, 28-day mortality, subgroup analysis

## Abstract

**Background:**

In recent years, septic shock remains a common fatal disease in the intensive care unit (ICU). After sufficient fluid resuscitation, some patients still experience tachycardia, which may lead to adverse effects on cardiac function. However, the use of β-blockers in the treatment of septic shock remains controversial. Thus, the purpose of this study is to evaluate the efficacy of β-blockers in the treatment of patients with septic shock and explore the most appropriate patient subgroups for this treatment.

**Methods:**

This retrospective observational study enrolled septic shock patients from the Medical Information Mart for Intensive Care (MIMIC)-IV and used propensity score matching (PSM) to balance some baseline differences between patients with and without β-blockers treatment. The primary outcome was the 28-day mortality. Length of stay (LOS) in the ICU and hospital, and the degree of support for organs such as circulatory, respiratory and renal systems were also assessed. Subgroup analysis and multivariate logistic regression were performed to determine the relationship between β-blockers therapy and 28-day mortality in different patient groups.

**Results:**

A total of 4,860 septic shock patients were enrolled in this study and 619 pairs were finally matched after PSM. Our analysis revealed that β-blocker therapy was associated with a significant improvement in 28-day mortality (21.5% vs. 27.1%; *P* = 0.020) and led to a prolonged LOS in both the ICU and hospital. Subgroup analysis indicated that there was an interaction between cardiovascular diseases and β-blocker therapy in patients with septic shock. Patients with pre-existing heart disease or atrial arrhythmias were more likely to derive benefits from β-blocker treatment.

**Conclusion:**

We found β-blockers therapy was effective to improve 28-day mortality in patients with septic shock. Patients in the subgroup with cardiovascular diseases were more likely to benefit from β-blockers in mortality.

## Background

In recent years, despite advancements in diagnostic and treatment technologies, septic shock remains a significant medical challenge in the intensive care unit (ICU), with mortality rates still ranging from 30% to 40% (1, 2). Septic shock often leads to multiorgan failure, and heart is one of the most frequently affected organs, which mainly characterized as impaired cardiac systolic and diastolic function without abnormal myocardial structure (3), and some studies have showed that patients with septic cardiomyopathy have a 2- to 3-fold higher mortality than patients without cardiomyopathy (4, 5). The specific mechanisms behind sepsis-induced cardiomyopathy remain unclear, but factors such as sympathetic overactivity (6) and compensatory tachycardia may contribute (7). β-blockers, which competitively antagonize catecholamines by binding to beta-adrenoreceptors primarily located in the heart, have been investigated as potential agents to mitigate cardiac toxicity in sepsis and septic shock. While some studies suggest that β-blockers may reduce myocardial injury and oxygen consumption in these patients (8, 9), their negative inotropic and chronotropic effects have raised concerns regarding their use in sepsis.

Animal models have shown that β-blockers can improve heart function (10), although there are conflicting results (11). Moreover, β-blockers have been found to improve mortality and maintain hemodynamic stability in patients who have received sufficient fluid resuscitation to eliminate compensatory tachycardia (12–14). In these clinical studies, although heart rate is controlled, there is no significant decrease in cardiac output. Conversely, some researchers have reported an exacerbation of septic shock, evident through elevated lactate levels or an increased dosage of vasoactive agents (9, 15).

The use of β-blockers in the treatment of sepsis and septic shock remains controversial, with limited research and small sample sizes contributing to the lack of clear guidelines or expert consensus on their indications. And Ge et al. (16) found that the effect of β-blockers on sepsis patients was consistent in septic shock, but not in non-septic shock. Therefore, this study selected a population with septic shock. Additionally, previous studies have inadequately explored subgroup analysis, potentially constrained by small sample sizes. Finally, this retrospective study utilized the MIMIC-IV database to analyze the potential benefits of β-blockers in patients with septic shock and further explored the most suitable treatment population through subgroup analysis.

## Materials and methods

### Data source

This retrospective observational study used the MIMIC-IV (version 1.0) database (17), which was developed and maintained by the Laboratory for Computational Physiology at MIT. MIMIC-IV database integrates comprehensive clinical information of patients in ICUs of the Beth Israel Deaconess Medical Center in Boston, Massachusetts, USA between 2008 and 2019. One author, Ling Zhang, passed the Collaborative Institutional Training Initiative Examination and obtained permission to extract data (certification number: 47869408). Due to the date collection in MIMIC-IV was performed with the institutional review board (IRB) approval of Massachusetts Institute of Technology and Beth Israel Deaconess Medical Center, IRB or local ethic committee approval was exempted in this study.

### Patient population

Firstly, we identified all adults experiencing their initial admission to the ICU with a length of stay (LOS) exceeding 48 h. Subsequently, individuals diagnosed with sepsis, as per the sepsis 3.0 criteria (18) underwent further screening. Among these, patients who received vasopressors (norepinephrine and vasopressin) 48 h before and after the sepsis diagnosis were categorized as having septic shock (19, 20). Exclusions were made for contraindications to β-blocker usage, such as acute myocardial infarction, acute heart failure, high-degree atrioventricular block (AVB), and asthma. The resulting group of septic shock patients comprised the target population for this study. The baseline time of enrollment was defined as the initial use of vasopressors, and patients were then stratified into two groups: the intervention group (those receiving β-blockers, including esmolol, metoprolol, and labetalol) and the control group (those not receiving β-blockers) based on β-blocker administration within 48 h after enrollment.

### Variable extraction

The following variables were extracted from the MIMIC IV database within 24 h after enrollment, with the most critical values considered for calculations: age, gender, weight, ICU types, heart rhythms, Sequential Organ Failure Assessment (SOFA) score, Simplified Acute Physiology Score II (SAPS II), Charlson comorbidity score, white blood cell count (WBC), hemoglobin (Hb), platelets, urea, creatinine, total bilirubin (TB), pH, oxygenation index (PaO2/FiO2), and lactate. Comorbidities such as hypertension and heart disease (including chronic heart failure, persistent atrial fibrillation, and coronary heart disease) were defined using ICD-9 or ICD-10 diagnosis codes. The use of vasopressors (including norepinephrine and vasopressin), mechanical ventilation, and renal replacement therapy (RRT) during the 28 days was also recorded. Basic heart rate (HR) and mean arterial pressure (MAP) were extracted according to the closest time of enrollment, while central venous pressure (CVP) was the mean value of 24 h. Also, data of HR and MAP within 48 h of enrollment were all extracted to evaluate the trend before and after intervention of β-blockers.

### Outcomes

The primary outcome was the 28-day mortality. The secondary outcomes included LOS in ICU and hospital, 28-day cumulative mechanical ventilation–free days, 28-day cumulative vasoactive agent–free days and 28-day cumulative RRT–free days.

### Statistical analysis

Continuous variables were presented as mean ± standard deviation and compared using the Student's *t*-test when the data were normally distributed. Non-normally distributed continuous variables were showed as median (interquartile range) and compared using the nonparametric Mann–Whitney *U*-test. Categorical variables were expressed as proportions and compared using the chi-squared test. To mitigate bias, propensity score matching (PSM) was performed to balance age, sex, weight, SOFA score, SAPS II, Charlson comorbidity score, heart disease and atrial arrhythmia between the two groups. Patients were matched using 1:1 nearest neighbor matching approach, ensuring that the standardized mean difference (SMD) of all aforementioned variables was ≤0.1. The multivariate logistic regression analysis was used to determine the relationship between β-blockers treatment and 28-day mortality. Confounding variables adjusted in the model were selected based on clinical relevance. Subgroup analyses for the primary outcome according to age, type of ICU, heart disease, hypertension, atrial arrhythmia, baseline mechanical ventilation and SOFA were also performed. A two-sided analysis with a *p*-value < 0.05 was considered statistically significant. All statistical analyses were conducted using IBM SPSS Statistics version 25.0 and R language version 3.6.1. Results were reported in the form of tables and graphs.

## Results

### Patients and baseline characteristics

**Figure 1:**
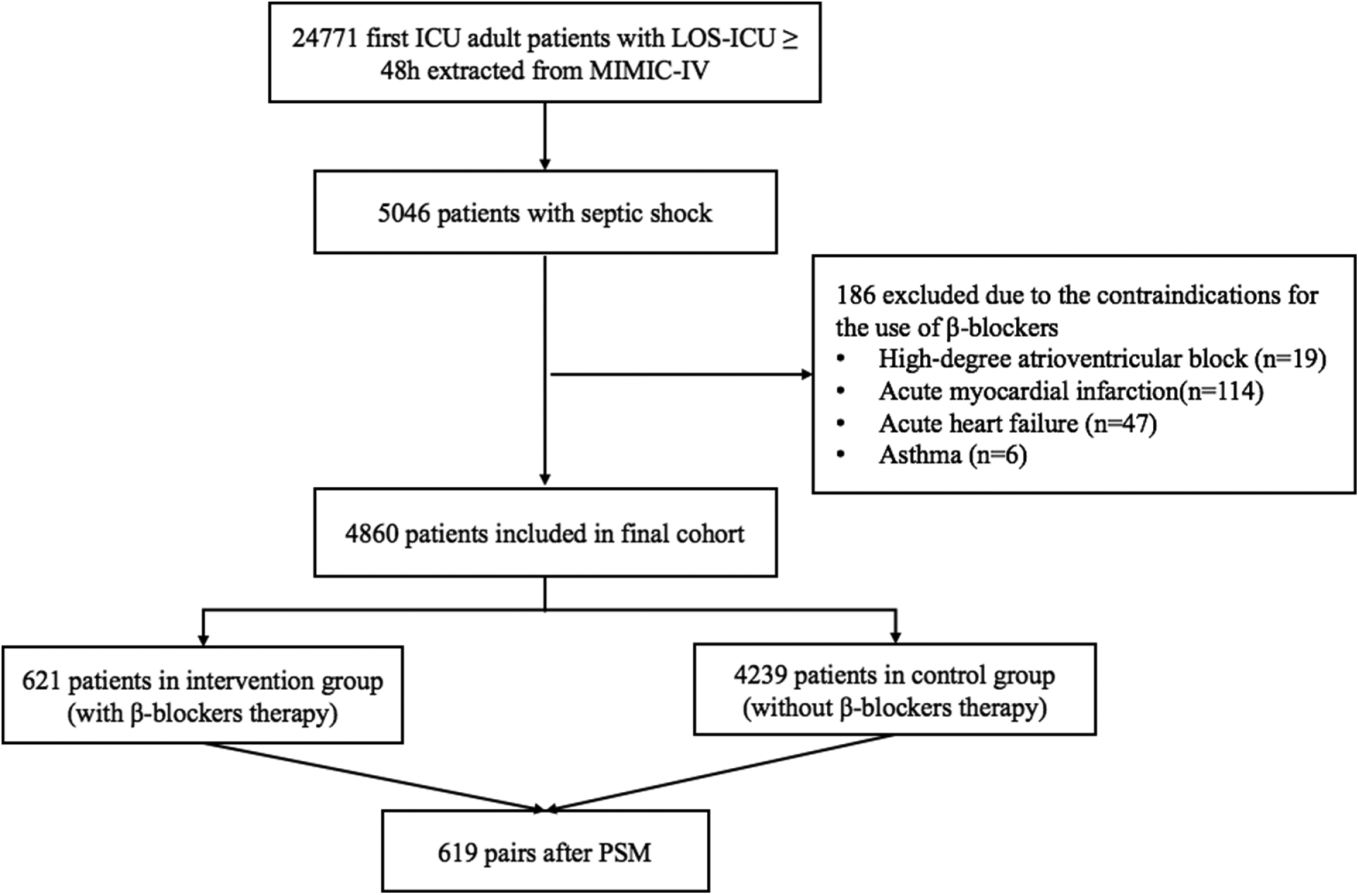
Flowchart of the patients. ICU, intensive care unit; LOS, length of stay; MIMIC-IV, medical information mart for intensive care IV; PSM, propensity score matching.

According to the inclusion and exclusion criteria, a total of 4,860 eligible septic shock patients from the MIMIC-IV database were enrolled in the final cohort. Among them, 621 patients administered β-blockers within 48 h of enrollment, while the remaining 4,239 patients did not. After achieving balance in age, sex, weight, SOFA, SAPS II, Charlson comorbidity score, heart disease and atrial arrhythmia between the two groups through PSM, 619 patients were included in the final analysis for each group. The detailed flowchart is shown in Figure 1.

Table 1 presents baseline demographics and clinical characteristics before and after PSM. Prior to PSM, the mean age of patients receiving β-blocker therapy was 72.4 years, while it was 67.3 years in the control group. The percentage of males was approximately 57% in both the intervention and control groups. Patients receiving β-blockers had a significantly higher body weight (83.0 vs. 80.0 kg; *P* = 0.039), faster baseline heart rate (94 vs. 91 bpm; *P* = 0.001), a higher proportion requiring RRT (6.8% vs. 11.6%; *P* < 0.001) and mechanical ventilation (78.6% vs. 72.7%; *P* = 0.002), and a higher SOFA score (9 vs. 10; *P* < 0.001), indicating greater disease severity compared to the control group. Additionally, the prevalence of heart disease and atrial arrhythmia was significantly higher in patients treated with β-blockers than in the control group.

**Table 1:** Baseline characteristics of patients before and after PSM.

Characteristics	Before PSM			After PSM		
	Intervention group (*N* = 621)	Control group (*N* = 4,239)	*P*	Intervention group (*N* = 619)	Control group (*N* = 619)	*P*
Age [median (IQR)], y	72.4 [62.9, 80.1]	67.3 [55.6, 78.0]	<0.001	72.4 [62.8, 80.1]	73.0 [61.2, 82.5]	0.389
Gender, male (%)	353 (56.8)	2,399 (56.6)	0.906	351 (56.7)	347 (56.1)	0.819
Weight [median (IQR)], kg	83.0 [68.3, 100.1]	80.0 [67.6–97.0]	0.039	83.0 [68.3, 100.1]	80.5 [68.7, 97.7]	0.328
Temperature [median (IQR)], °C	37.6 [37.1, 38.2]	37.4 [37.1, 38.1]	0.009	37.6 [37.1, 38.2]	37.3 [37.0, 38.0]	<0.001
HR [median (IQR)], bpm	94 [80, 110]	91 [78, 107]	0.001	94 [80, 110]	90 [78, 105]	0.001
MAP [median(IQR)], mmHg	66 [57, 76]	65 [56, 75]	0.075	66 [57, 76]	64 [55, 74]	0.027
CVP [median(IQR)], mmHg	13.2 [10.1, 17.4]	12.7 [9.4, 17.5]	0.196	13.2 [10.1, 17.4]	12.2 [9.8, 16.6]	0.095
ICU types, (%)			<0.001			<0.001
MICU	207 (33.3)	2,573 (41.1)		205 (33.1)	312 (50.4)	
SICU	194 (31.2)	1,496 (23.9)		194 (31.3)	136 (22.0)	
CCU	205 (33.0)	2,098 (33.5)		205 (33.1)	165 (26.7)	
NSICU	15 (2.4)	93 (1.5)		15 (2.4)	6 (1.0)	
Heart disease, (%)	230 (37.0)	1,310 (30.9)	0.002	230 (37.2)	252 (40.7)	0.200
Atrial arrhythmia, (%)	86 (13.8)	166 (3.9)	<0.001	86 (13.9)	84 (13.6)	0.869
Ventricular arrhythmia, (%)	3 (0.5)	7 (0.2)	0.126	3 (0.5)	1 (0.2)	0.624
RRT, (%)	42 (6.8)	492 (11.6)	<0.001	41 (6.6)	65 (10.5)	0.015
Mechanical ventilation, (%)	488 (78.6)	3,080 (72.7)	0.002	486 (78.5)	409 (66.1)	<0.001
SAPSII [median (IQR)]	46 [38, 56]	46 [37, 57]	0.423	46 [38, 56]	46 [38, 56]	0.830
SOFA [median (IQR)]	9 [7, 12]	10 [8, 13]	<0.001	9 [7, 12]	9 [7, 12]	0.749
Charlson comorbidity score [median (IQR)]	6 [5, 8]	6 [4, 8]	0.060	6 [5, 8]	6 [5, 8]	0.299
WBC, × 10^9^/L	15.0 [10.8, 20.4]	14.9 [10.3, 21.0]	0.499	15.0 [10.8, 20.5]	15.0 [10.3, 20.2]	0.338
Hb, g/dl	9.5 [8.4, 11.0]	9.4 [8.2, 10.9]	0.088	9.5 [8.4, 11.0]	9.3 [8.3, 10.7]	0.067
Platelets, × 10^9^/L	147 [103, 207]	150 [96, 221]	0.676	147 [103, 208]	163 [106, 239]	0.006
Urea, mg/dl	26 [18, 42]	29 [18, 47]	0.030	26 [18, 41]	29 [18, 45]	0.061
Creatinine, mg/dl	1.3 [0.9, 2.2]	1.4 [0.9, 2.5]	0.011	1.3 [0.9, 2.2]	1.4 [0.9, 2.4]	0.219
TB, mg/dl	0.9 [0.6, 1.9]	1.0 [0.5, 2.7]	0.089	0.9 [0.6, 1.9]	0.8 [0.5, 1.9]	0.186
PH	7.31 [7.25, 7.36]	7.30 [7.21, 7.36]	<0.001	7.31 [7.25, 7.36]	7.31 [7.23, 7.36]	0.378
PaO_2_/FiO_2_	156 [98, 240]	123 [80, 204]	<0.001	156 [98, 240]	142 [88, 216]	0.022
Lactate, mmol/L	2.5 [1.6, 4.2]	2.4 [1.5, 4.3]	0.376	2.5 [1.6, 4.2]	2.3 [1.5, 4.0]	0.031

Data are presented as mean ± SD or median (interquartile range) for skewed variables or proportions for categorical variables.

PSM, propensity score matching; IQR, interquartile range; HR, heart rate; MAP, mean arterial pressure; CVP, central venous pressure; ICU, intensive care unit; MICU, medical intensive care unit; SICU, surgical intensive care unit; CCU, cardiac care unit; NSICU, neurosurgical intensive care unit; RRT, renal replacement therapy; SAPS II, simplified acute physiology score II; SOFA, sequential organ failure assessment; WBC, white blood cell; Hb, hemoglobin; TB, total bilirubin.

After PSM, the differences between the two groups in age, sex, weight, SOFA, SAPS II, Charlson comorbidity score, heart disease and atrial arrhythmia were eliminated. However, in terms of vital signs, the intervention group still exhibited higher baseline HR (94 vs. 90 bpm; *P* = 0.001), temperature (37.6℃; vs. 37.3℃; *P *< 0.001), and MAP (66.0 vs.63.5 mmHg; *P* = 0.027) than the control group. As for the ICU subgroup, the ratio of NSICU in both groups was less than 5%, which was relatively small. Then, patients in the intervention group were evenly distributed in MICU, SICU, and CCU, while in the control group, more than half of the patients came from MICU, which was significantly different from the intervention group. Moreover, baseline laboratory data including WBC, Hb, urea, creatinine, TB and PH were no statistically significant differences between two groups. But the intervention group had lower platelet count (147 vs. 163 × 10^9^/L; *P* = 0.006) and higher lactate level (2.5 vs. 2.3 mmol/L; *P* = 0.031), both of which were statistically significant.

### Association between β-blockers treatment and hemodynamic variables

**Figure 2:**
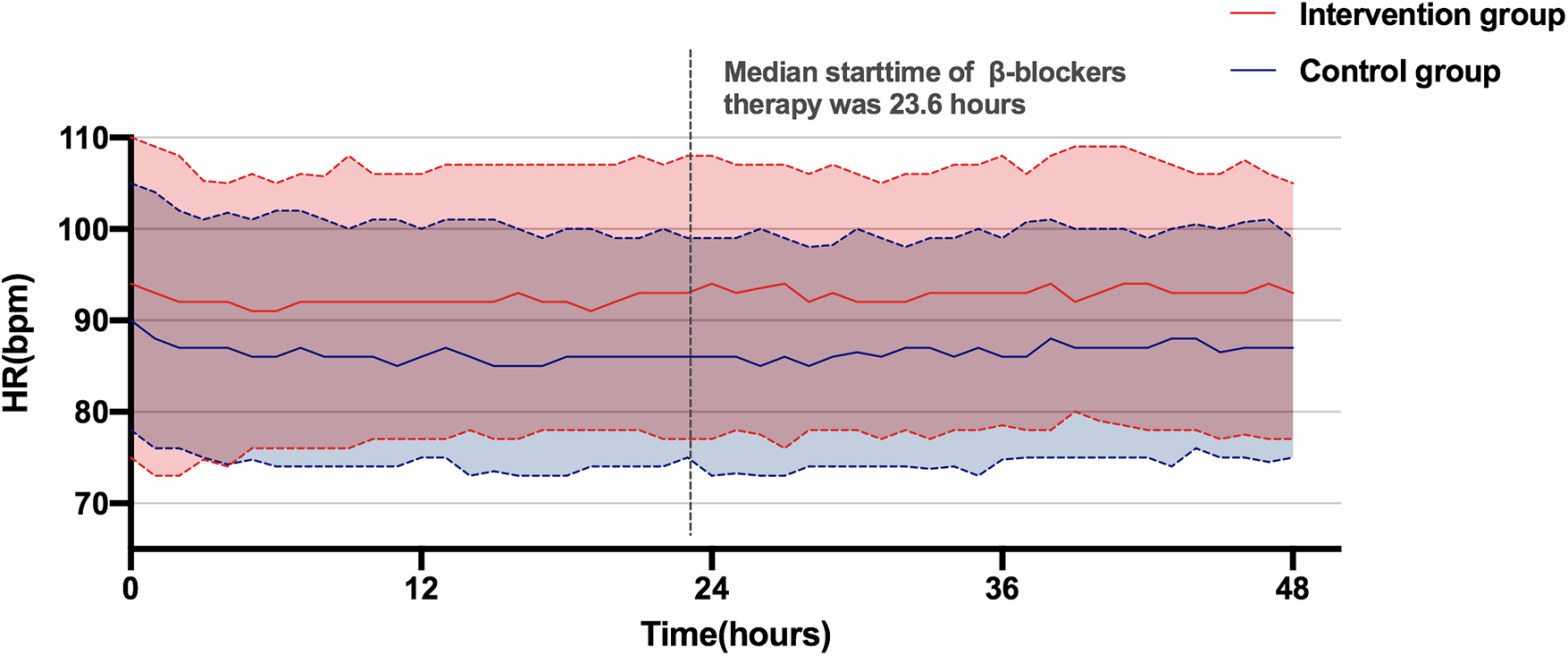
Changes in heart rate within 48 h after enrollment. HR, heartrate.

Both Figure 2 and Table 2 show the changes in hemodynamics including HR and blood pressure after β-blocker therapy. Figure 2 presents the hourly trends in HR over 48 h post-enrollment for both groups. It reveals that the average HR of the control group remained below 90 bpm, while HR fluctuated around 95 bpm in the intervention group. The median initiation time of β-blocker intervention was 23.6 h after enrollment, and the gap in HR between the two groups didn't widen beyond this point. Table 2 shows the HR and blood pressure values before and after treatment. Prior to β-blocker therapy, the average HR for all patients in the intervention group was 94.1 bpm, increasing to 95.9 bpm during the intervention period up to 48 h post-enrollment, with no significant changes observed. Moreover, there were no substantial fluctuations in the average MAP following β-blocker treatment, which was 74.4 mmHg before intervention and 75.8 mmHg after.

**Table 2:** Hemodynamic variables of patients before and after β-blockers therapy.

Characteristics	Intervention group (*N* = 619)		Control group (*N* = 619)
	Before β-blockers therapy	After β-blockers therapy	
HR [median (IQR)], bpm	94.1 [82.7, 107.3]	95.9 [85.3, 106.1]	87.0 [76.5, 98.0]
MAP [median (IQR)], mmHg	74.4 [68.6, 80.6]	75.8 [70.8, 83.7]	71.9 [67.8, 76.9]

Data are presented as mean ± SD or median (interquartile range) for skewed variables or proportions for categorical variables.

IQR, interquartile range; HR, heart rate; MAP, mean arterial pressure.

### Primary and secondary outcomes

The primary outcome of 28-day mortality was 21.5% in the intervention group and 27.1% in the control group (*P* = 0.020). Additionally, in terms of secondary outcomes, we observed that the intervention group had a longer LOS in ICU (5.92 vs. 5.04 days; *P* = 0.001) and hospital (12.36 vs. 11.29 days; *P* = 0.018) compared to the control group. Furthermore, other secondary outcomes such as 28-day cumulative mechanical ventilation-free days was statistically reduced in the intervention group, while 28-day cumulative vasoactive agent-free days was increased. However, the 28-day cumulative RRT-free days were similar between the two groups (Table 3).

**Table 3:** Outcomes of patients.

Outcomes	Intervention group (*N* = 619)	Control group (*N* = 619)	*P*
Primary outcome			
28-day mortality, no. (%)	133 (21.5)	168 (27.1)	0.020
Secondary outcomes			
LOS [median (IQR)], days			
In ICU	5.92 [3.64, 10.92]	5.04 [3.23, 9.23]	0.001
In hospital	12.36 [7.86, 20.46]	11.29 [6.81, 18.62]	0.018
28-day cumulative mechanical ventilation–free days [median (IQR)], days	26.23 [22.23, 27.54]	26.60 [23.42, 28.00]	0.002
28-day cumulative vasoactive agent–free days [median (IQR)], days	26.75 [24.95, 27.56]	26.10 [24.30, 27.17]	<0.001
28-day cumulative RRT–free days [median (IQR)], days	28.00 [28.00, 28.00]	28.00 [28.00, 28.00]	0.685

Data are presented as mean ± SD or median (interquartile range) for skewed variables or proportions for categorical variables.

LOS, length of stay; IQR, interquartile range; ICU, intensive care unit; RRT, renal replacement therapy.

### Subgroup analysis

**Figure 3:**
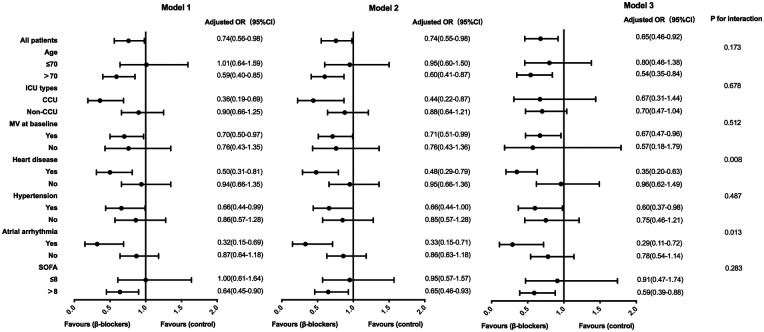
Association between β-blockers treatment and 28-day mortality in subgroups. Model 1: adjusted by basic heart rate, mean arterial pressure and temperature. Model 2: further adjusted by mechanical ventilation and renal replacement therapy on model 1. Model 3: further adjusted by platelets, PaO2/FiO2 and lactate on model 2. OR, odds ratio; CI, confidence interval; ICU, intensive care unit; CCU, cardiac care unit; MV, mechanical ventilation; SOFA, sequential organ failure assessment.

In a subgroup analysis according to age, ICU type, baseline mechanical ventilation, heart disease, hypertension, atrial arrhythmia and SOFA, the effect of β-blocker therapy on 28-day mortality was significantly associated with cardiovascular diseases such as heart disease and atrial arrhythmias (*P* for interaction < 0.05). Following multivariate logistic regression analysis, where variables such as baseline HR, MAP, temperature, mechanical ventilation, RRT, platelet count, PaO2/FiO2 ratio, and lactate levels were further adjusted, 28-day mortality of patients with pre-existing heart disease [OR 0.35 (95%CI 0.20–0.63)] or atrial arrhythmias [OR 0.29 (95%CI 0.11–0.72)] was significantly decreased after using β-blocker (Table 4, Figure 3).

**Table 4:** Subgroup analysis for 28-day mortality according to baseline.

Subgroup	Intervention group		Control group		*P*
	No. of patients	28-day mortality, no. (%)	No. of patients	28-day mortality, no. (%)	
Age, year					
≤70	265	57 (21.5)	272	56 (20.6)	0.793
>70	354	76 (21.5)	347	112 (32.3)	0.001
ICU types					
CCU	205	29 (14.1)	165	43 (26.1)	0.004
Non-CCU	414	104 (25.1)	454	125 (27.5)	0.421
MV at baseline					
Yes	486	107 (22.0)	409	119 (29.1)	0.015
No	316	40 (12.7)	448	63 (14.1)	0.576
Heart disease					
Yes	230	39 (17.0)	252	74 (29.4)	0.001
No	389	94 (24.2)	367	94 (25.6)	0.645
Hypertension					
Yes	352	63 (17.9)	315	81 (25.7)	0.014
No	267	70 (26.2)	304	87 (28.6)	0.521
Atrial arrhythmia					
Yes	86	16 (18.6)	84	35 (41.7)	0.001
No	533	117 (22.0)	535	133 (24.9)	0.262
SOFA					
≤8	254	42 (16.5)	255	43（16.9）	0.921
>8	365	91 (24.9)	364	125(34.3)	0.005

Data are presented as proportions for categorical variables.

ICU, intensive care unit; CCU, cardiac care unit; MV, mechanical ventilation; SOFA, sequential organ failure assessment.

Further analysis was conducted on patients with and without heart disease to evaluate the impact of β-blockers on outcomes. We found that patients with heart disease had a significant reduction in 28-day mortality after using β-blockers, but this benefit was not observed in patients without heart disease. Although 28-day cumulative vasoactive agent–free days was both increased in patients with and without heart disease after β-blockers therapy, this improvement in circulation seemed to be more prominent in patients with heart disease (Table 5).

**Table 5:** Outcomes of patients with and without heart disease.

Outcomes	With heart disease			Without heart disease		
	Intervention group (*N* = 230)	Control group (*N* = 252)	*P*	Intervention group (*N* = 389)	Control group (*N* = 367)	*P*
Primary outcome						
28-day mortality, no. (%)	39 (17.0)	74 (29.4)	0.001	94 (24.2)	94 (25.6)	0.645
Secondary outcomes						
LOS [median (IQR)], days						
In ICU	5.88 [3.68, 10.18]	4.99 [3.21, 8.51]	0.021	5.92 [3.53, 11.33]	5.12 [3.23, 9.52]	0.028
In hospital	12.42 [8.52, 18.35]	11.70 [6.86, 17.02]	0.112	12.24 [7.34, 21.90]	11.00 [6.75, 19.81]	0.079
28-day cumulative mechanical ventilation–free days [median (IQR)], days	26.45 [22.54, 27.55]	27.00 [24.13, 28.00]	0.011	25.88 [21.75, 27.54]	26.35 [22.86, 28.00]	0.052
28-day cumulative vasoactive agent–free days [median (IQR)], days	26.96 [25.46, 27.58]	25.90 [24.02, 27.11]	<0.001	26.65 [24.54, 27.53]	26.25 [24.48, 27.23]	0.006
28-day cumulative RRT–free days [median (IQR)], days	28.00 [28.00, 28.00]	28.00 [28.00, 28.00]	0.375	28.00 [28.00, 28.00]	28.00 [28.00, 28.00]	0.220

Data are presented as mean ± SD or median (interquartile range) for skewed variables or proportions for categorical variables.

LOS, length of stay; IQR, interquartile range; ICU, intensive care unit; RRT, renal replacement therapy.

## Discussion

Our retrospective analysis of the MIMIC-IV database revealed that β-blocker therapy was associated with improvements in 28-day mortality and prolonged LOS in both ICU and hospital among patients with septic shock. These findings align with conclusions drawn from previous observational and randomized controlled trials (RCTs) (8, 16). Furthermore, our subgroup analysis indicated that patients with cardiovascular diseases (such as pre-existing heart disease and atrial arrhythmias) were more likely to benefit from β-blocker therapy.

Septic shock triggers a potent activation of the sympathetic-adrenomedullary system, leading to a massive release of catecholamines into the bloodstream, reaching levels tens or even hundreds of times higher than normal. These hormones bind to α-receptors and β-receptors in the heart, blood vessels, and other organs, inducing vasoconstriction, increased heart rate, and augmented cardiac contractions to sustain tissue perfusion (21). The heightened heart rate observed during shock can be categorized into compensatory and non-compensatory phases. Prolonged tachycardia following adequate fluid resuscitation signifies a non-compensatory state, potentially reflecting sympathetic overactivity. Prolonged exposure to excessive stress may culminate in myocardial cell damage, necrosis, and myofibroblastic proliferation (22). Tachycardia may elevate oxygen consumption and shorten diastolic time, compromising myocardial blood supply and contributing to septic cardiomyopathy, marked by arrhythmias, impaired cardiac systolic and diastolic function, and elevated myocardial enzymes (23). Moreover, inappropriate tachycardia can downregulate catecholamine receptors, possibly reducing the responsiveness to catecholamine therapy (24). Persistent elevation in heart rate has been correlated with an increased incidence of cardiac events (25) and decreased survival rates (26). Theoretically, β-blockers competitively antagonize catecholamines by binding to β-receptors predominantly in the heart. Consequently, β-blocker administration may suppress sympathetic excitation, lower heart rate, and diminish myocardial oxygen consumption, offering potential benefits for patients experiencing excessive stress. Additionally, some studies suggest that β-blockers can attenuate levels of inflammatory factors and modulate immune and metabolic functions (14). As a result, over the past decade, β-blockers have been investigated as potential treatments for severe sepsis or septic shock, yielding promising results in several studies.

Recent meta-analyses (12, 14) have highlighted the potential benefits of β-blockers for patients with sepsis or septic shock who exhibit persistent tachycardia despite adequate fluid resuscitation. These benefits primarily involve ventricular rate control and a reduction in 28-day mortality. However, conflicting results have been reported in some studies, with suggestions of reduced tissue perfusion (9, 15). Discrepancies among these studies may stem from factors such as small sample sizes, heterogeneity in patient populations, and variability in β-blocker intervention protocols. In our study, we aimed to address these limitations by targeting similar patient populations. The median initiation time of β-blocker therapy in our study was 23.6 h after the diagnosis of septic shock, aligning with the fluid optimization or stabilization stage as recommended in sepsis bundles. Additionally, both groups in our study had achieved CVP of 12 mmHg within 24 h post-enrollment, indicating adequate volume status. Furthermore, the median baseline heart rate in both groups exceeded 90 bpm, suggesting persistent tachycardia. In contrast to previous studies, our research included a larger sample size, exceeding 1,000 patients, thereby enhancing statistical power. Moreover, we employed PSM to further balance baseline characteristics between the two groups and mitigate the influence of confounding variables. Consistently, our findings demonstrated a significant reduction in 28-day mortality in the intervention group (21.5%) compared to the control group (27.1%). Furthermore, subgroup analysis revealed that patients with cardiovascular diseases (including pre-existing heart disease and atrial arrhythmias) experienced a lower 28-day mortality in the intervention group compared to the control group. This finding suggests that individuals with cardiac comorbidities may be particularly susceptible to adverse cardiovascular events during sustained tachycardia, thereby highlighting the importance of β-blocker therapy in this population. Sander et al. (25) shown that incidence of major cardiac events in cardiac high-risk patients with prolonged elevated heart rates was 49%, significantly higher than 13% in the control group. Previous studies have demonstrated the efficacy of β-blockers in controlling heart rate and reducing mortality in patients with conditions such as acute myocardial infarction and chronic heart failure (27, 28). In high-risk cardiac patients, prolonged elevation in heart rate has been associated with a significantly increased incidence of major cardiac events. Therefore, the use of β-blockers during perioperative periods or periods of stress can effectively suppress catecholamine surges and mitigate cardiovascular events (29).

While research on the use of β-blockers in sepsis and septic shock is increasing, consensus regarding specific treatment protocols, such as the timing of drug intervention, remains elusive. Existing studies have varied in their criteria for selecting intervention subjects, particularly in the assessment of persistent tachycardia despite fluid resuscitation. Some studies (8) relied on CVP thresholds, such as CVP ≥8 mmHg, as an indicator of sufficient volume status, a criterion which we also adopted in our research. However, other studies have evaluated fluid responsiveness through repeated fluid challenges (9), a method deemed safer and more effective, and one which could potentially be standardized in future research efforts. As for the start time of intervention, Morelli et al. (8) administered esmolol in patients who were still using norepinephrine after 24 h of hemodynamic optimization and reported its improvement in mortality. While Levy et al. (15) chose early esmolol use on patients treated with norepinephrine for a minimum of 6 h and average of 9 h, finding that patients couldn't benefit from using β-blockers and even had an increased risk of hypotension. In our study, median start time of β-blockers using was 23.6 h after diagnosis of septic shock, between the above two studies. We speculate that initiating treatment too early may lead to hemodynamic fluctuations, as the body may not have stabilized yet. Conversely, delaying intervention may prolong exposure to catecholamines and delay the effects of sympathetic excitation inhibition. Therefore, further research is warranted to determine the optimal timing for initiating β-blocker therapy in patients with septic shock. Standardized protocols for assessing fluid responsiveness and consistent criteria for identifying patients who may benefit from β-blocker therapy are also needed to advance our understanding and optimize treatment strategies in this patient population.

The optimal goal of β-blocker therapy in sepsis and septic shock is to control HR to reduce myocardial oxygen consumption while maintaining cardiac output and tissue perfusion. However, determining the ideal heart rate target is crucial, and existing approaches typically rely on selecting patients with tachycardia (HR ≥ 95 bpm or 100 bpm) for intervention, aiming to maintain the heart rate between 80 bpm and 94 bpm or decrease it by 10%–20%. And, in this study, the median HR was controlled at around 95 bpm through the use of blockers in the intervention group. However, this target range is often based on empirical experience rather than robust evidence-based medicine, and there is inherent baseline heterogeneity in each patient's heart rate, which may limit the effectiveness of treatment. In light of these challenges, dynamic evaluation of hemodynamics may offer valuable insights. Studies reporting improved mortality with β-blocker therapy have observed stable cardiac index (CI) and stroke volume (SV) following treatment (8). Conversely, studies with negative results or an increased trend in mortality have noted a decrease in CI or an escalation in the dosage of vasoactive drugs (15). Therefore, dynamic hemodynamic monitoring could serve as a guide for β-blocker treatment, facilitating the titration of the optimal dose. Adopting a goal-directed therapy approach based on dynamic hemodynamic monitoring may enhance safety and mitigate adverse events such as decreased cardiac output and inadequate tissue perfusion, which have been central controversies surrounding β-blocker therapy in sepsis and septic shock. By tailoring treatment to individual patient responses, this approach has the potential to improve outcomes and address the variability observed in patient responses to β-blocker therapy.

Our study has several limitations. Firstly, this was a retrospectively study, which may introduce inherent biases and limit the generalizability of our findings. Despite efforts to mitigate this through PSM, differences between groups may still exist. Secondly, the data from the MIMIC database spanned over 10 years, the treatment strategies for septic shock may have changed during this period, which may affect the results. Thirdly, there were variations in the timing of initiating β-blocker therapy in the intervention group, and the lack of a standardized HR target goal hinders the comprehensive evaluation of the therapeutic effect. Fourthly, though β-blockers extracted from MIMIC-IV database contained esmolol, metoprolol and labetalol, a long-acting β-adrenergic receptor antagonist metoprolol accounts for nearly 90%, making it difficult to dynamically adjust dosage to titrate heart rate to the target value in a short time. Finally, incomplete documentation of patients’ medication histories, including prior β-blocker use, may introduce confounding factors that could influence the study outcomes.

## Conclusion

This study demonstrates that β-blocker therapy in septic shock patients is associated with improved 28-day mortality and prolonged LOS in the ICU and hospital compared to those not receiving β-blockers. Subgroup analysis further reveals a significant reduction in 28-day mortality among patients with cardiovascular diseases. In conclusion, β-blockers show promise in treating septic shock patients post-adequate fluid resuscitation. However, further large-scale RCTs are needed to confirm these findings and explore optimal initiation and cessation times for intervention, as well as identify the most suitable treatment subgroups and target goals. These efforts will enhance our understanding of the role of β-blockers in the management of septic shock and inform more precise treatment strategies for improving patient outcomes.

## Data Availability

The original contributions presented in the study are included in the article/Supplementary Material, further inquiries can be directed to the corresponding authors.
